# Dexamethasone Preconditioning in Cardiac Procedures Reduces Decreased Antithrombin Activity and Is Associated to Beneficial Outcomes: Role of Endothelium

**DOI:** 10.3389/fphar.2018.01014

**Published:** 2018-09-25

**Authors:** Vicente Muedra, Lucrecia Moreno, Vicente Rodilla, Cristina Arce, Fermi Montó, Águeda Blázquez, Paloma Pérez, Pilar D’Ocón

**Affiliations:** ^1^Departamento de Anestesiología, Cuidados Críticos y Terapéutica del Dolor, Hospital Universitario de La Ribera, Valencia, Spain; ^2^Departamento de Cirugía, Facultad de Ciencias de la Salud, Universidad CEU Cardenal Herrera, Valencia, Spain; ^3^Departamento de Farmacia, Facultad de Ciencias de la Salud, Universidad CEU Cardenal Herrera, Valencia, Spain; ^4^Departamento de Farmacología, Facultad de Farmacia, Universitat de València, Valencia, Spain; ^5^Estructura de Recerca Interdisciplinar en Biotecnologia i Biomedicina (ERI BIOTECMED), Universitat de València, Valencia, Spain; ^6^Instituto de Biomedicina de Valencia-Consejo Superior de Investigaciones Científicas (IBV-CSIC), Valencia, Spain

**Keywords:** angiogenesis, antithrombin, cardiac surgery, cardiopulmonary bypass, dexamethasone, endothelial function

## Abstract

**Introduction:** Decreased antithrombin (AT) activity in patients scheduled for cardiovascular surgery under cardiopulmonary bypass (CPB) is related to increased postoperative complications and hospitalization time. Indirect evidence suggests that glucocorticoids mitigate this decreased AT activity. To better understand the beneficial effects of AT we have analyzed: (i) the clinical relevance of acute dexamethasone (DX) administration before cardiac surgery on AT activity, (ii) the modulation by DX of AT expression in human endothelial cells (hECs), (iii) the activity of AT on migration and angiogenesis of hECs, or on angiogenesis of rat aorta.

**Methods:** A retrospective cohort study in patients undergoing aortic valve replacement surgery was designed to evaluate the effect of DX administration on AT activity at five separate time points: preoperatively, during CPB, at intensive care unit admission and at 12 and 24 h post-intervention. We have analyzed also clinical differences in postoperative outcomes as safety and the length of stay in hospitalization. Changes in mRNA levels of AT induced by DX were determined by qRT-PCR in human coronary (hCEC), aorta (hAEC) and cardiac microvasculature (hCMEC) endothelial cells. AT activity on migration and angiogenesis were also assayed. Angiogenic growth of rat aortic rings incubated in Matrigel^®^ was determined in presence and absence of AT.

**Results:** The cohort comprised 51 patients in the control group and 29 patients in the group receiving dexamethasone. Preoperative DX supplementation reduced intraoperative decrease of AT activity (67.71 ± 10.49% DX treated *vs.* 58.12 ± 9.11% untreated, *p* < 0.001) that could be related to a decrease in the hospitalization time (7.59 ± 4.08 days DX treated *vs.* 13.59 ± 16.00 days untreated, *p* = 0.014). Treatment of hECs with 500 nM DX slightly increased AT expression. Incubation with 0.5 and 1 IU/mL of AT increased migration and angiogenesis in hCAECs and hAECs, but not in hCMECs. The same concentrations of AT potentiated angiogenic sprouting of new vessels from rat aorta.

**Conclusion:** Preoperative DX supplementation could be an interesting procedure to avoid excessive decrease in AT levels during cardiac surgery. Positive outcomes associated with maintaining adequate AT levels could be related to its potential beneficial effect on endothelial function (migration and angiogenesis).

## Introduction

Antithrombin (AT) is a serin-protease inhibitor encoded by *SERPINC1* gene, which belongs to the serpin family (Ser-protease inhibitor, clade C, member 1). AT is a plasma protein, synthesized primarily in the liver (Kourteva et al., 1995), but which is also expressed to a lesser extent, in kidneys, platelets and endothelial cells (Chan and Chan, 1981; Kourteva et al., 1995). As the major anti-coagulant protein, AT inhibits the action of thrombin and other proteases (Gierer et al., 2013) and probably has specific anti-inflammatory properties and a protective endothelial action (Travis and Salvesen, 1983; Huntington, 2003).

Endothelium activation is considered a key process in the development of the inflammatory response secondary to CPB since it plays an important role in the regulation of vascular tone and synthesis of thrombo-regulatory substances (Schmid et al., 2006). In addition the endothelium is an active reservoir of essential plasma proteins (i.e., albumin, heparan-sulfate, antioxidants and AT) (Myers et al., 2017). Experimental studies have revealed that serine proteases, such as thrombin, lead to loss of hyaluronan from the glycocalyx, thus suggesting a wide array of potentially destructive conditions. Pharmacological agents such as inhibitors of inflammation, metallo-proteases and AT display potential to attenuate shedding of this endothelial surface layer (Becker et al., 2015).

Endothelial dysfunction is a risk for ischemic events such a stroke, myocardial infarction, unstable angina pectoris, ventricle fibrillation, need for revascularisation procedures and death from cardiovascular reasons. Clinical studies have shown that cardiac surgery has an impact on vascular endothelial function (Verrier and Morgan, 1998).

In a clinical setting, decreased AT levels have been related to a number of events by consumption, deficiency or other procoagulant situations (van Ommen and Nowak-Göttl, 2017). Severe deficiency of AT, hence increasing the risk of thrombotic event has been associated with antineoplastic agent such as a L-asparaginase, estrogen-containing contraceptives (Hernández-Espinosa et al., 2009) or genetic defects on exons or flanking regions of *SERPINC1* (Toderici et al., 2016), increase the risk of thrombotic events. AT deficiency in the early postoperative period following major surgery (Duswald et al., 1985; Jordan et al., 1987; Matsutani et al., 1998) has also been associated with increased morbidity and mortality (Fourrier et al., 1992; Hasegawa et al., 1994; Ranucci et al., 1999, 2005; Paparella et al., 2009; Muedra et al., 2013a,b).

During cardiac surgery with CPB, the levels of AT activity decrease owing to consumption (Ranucci et al., 2004) and hemodilution (Linden et al., 2004), resulting in a deficient amount of this protein at the end of the procedure, which together with high thrombin generation may trigger postoperative thromboembolic complications (Hashimoto et al., 1994; Slaughter et al., 2001). Poor response to heparin, commonly defined as heparin resistance, which occurs at a variable rate between 10 and 30% has been associated with low plasma levels of AT before and during these cardiac procedures (Ranucci et al., 1999, 2005; Muedra et al., 2013a). Some observational studies have shown that an inverse relationship between decreased postoperative AT plasmatic levels and complication incidence exists. Complications include thromboembolic events, adverse neurologic events, bleeding and prolonged intensive care unit stay after cardiac operations, need for mechanical ventilation, more surgical re-explorations and blood transfusions (Ranucci et al., 2005; Paparella et al., 2009; Garvin et al., 2010; Muedra et al., 2011; Muedra et al., 2013a). All these complications related to decreased AT activity, could result in a marked increase in the postoperative costs (Muedra et al., 2013a).

Paparella et al. (2009) showed that after admission to ICU, patients with AT activity levels <63.7% had a much greater incidence of adverse cardiac events, such as excessive bleeding, infections, stroke, acute renal failure, sepsis and atrial fibrillation. Recently, Ranucci et al. (2013) defined a cut-off value of AT activity at arrival in the ICU (<58%) as the most specific and sensitive value to predict a prolonged ICU stay.

Failure to achieve an acceptable AT level is usually managed by the administration of AT (concentrate purified or recombinant) or fresh frozen plasma as a source of AT in an attempt to restore heparin responsiveness (Lemmer and Despotis, 2002; Avidan et al., 2005; Ranucci et al., 2005). Furthermore, there are guidelines that recommend AT supplementation for the prevention of thromboembolic complications for selected patients (Society of Thoracic Surgeons Blood Conservation Guideline Task Force: Ferraris et al., 2011). However, AT administration is not risk-free, as it has been linked to transfusion-related acute lung injury, viral infections and allergic reactions (Kanbak, 1999). A high bleeding risk could be produced in cases of indiscriminate AT supplementation (Ranucci et al., 2013). Therefore, an equilibrium in AT levels must be obtained to avoid postoperative complications, AT supplementation in cardiac operations with cardiopulmonary bypass (CPB) is still an open issue.

A new therapeutic target could be the pharmacological modulation of endogenous AT synthesis as this would provide an alternative to purified (or recombinant) AT supplementation. This strategy could be more efficient than exogenous supplementation (Barettino et al., 2015) to avoid the AT overcorrection observed (Ranucci et al., 2013). Following this assumption, we analyzed the regulatory elements present in the *SERPINC1*, encoding AT. *SERPINC1* promoter region is relatively simple with regulatory elements for a correct hepatic expression being located 700 bp upstream of the transcription start site (Tremp et al., 1995; Fernandez-Rachubinski et al., 1996). One of the important regulatory elements in the *SERPINC1* promoter is a Hormonal Regulatory Element (HRE) for Nuclear Hormone Receptors (NHRs), with a complex structure overlapping hepatocyte nuclear factor 4 binding sites (Niessen et al., 1996). Our preliminary results showed that several compounds activating NHRs were able to increase the expression of *SERPINC1* in human HepG2 hepatoma cells. In particular, retinoids activating the Retinoid X Receptor (RXR), glucocorticoids (methyl-prednisolone, cortisone or dexamethasone) and the Farnesoid X Receptor (FXR) ligand GW4064 appeared as the best activators of AT expression (Barettino et al., 2015).

Preconditioning with Dexamethasone (DX) can be easily translated to clinical practice and additionally would have an impact on health care costs which would be significantly lower than other pharmacological options. DX could be a potential therapeutic alternative to mitigate the decreased AT activity in cardiac surgery and thus pre-condition the patient against adverse effects and postoperative complications.

To find out the clinical relevance of this modulation by DX, a preliminary observational study has been conducted in patients enrolled in an enhanced recovery after surgery (ERAS) program for cardiac procedures, in which DX was initially included to prevent postoperative nausea and vomiting, therefore facilitating the patient early recovery.

In parallel, an experimental study was designed to evaluate AT activity at the vascular endothelium, by analyzing AT expression on human aortic endothelial cells (hAEC), coronary artery (hCAEC) and cardiac microvasculature (hCMEC) and its modulation by DX, as well as studying the regulation of migration and angiogenesis, to explore potential mechanisms that would justify the benefits of therapeutic AT in cardiac surgery.

## Materials and Methods

### Clinical Studies

#### Study Design

A preliminary retrospective cohort study was designed to evaluate the efficacy of DX, avoiding excessive postoperative decrease of AT activity in patients undergoing aortic valve replacement surgery, as a primary endpoint. Additionally, the study aimed to determine clinical differences in postoperative outcomes, safety and hospitalization time.

#### Study Population

**FIGURE 1:**
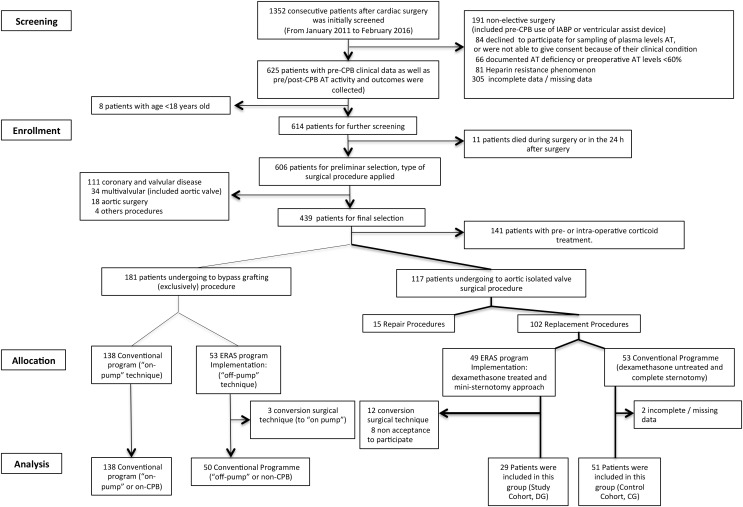
Flow diagram for the patient selection process.

From January 2011 to February 2016 patients undergoing cardiac surgery were considered for enrolment at a single center (La Ribera University Hospital, Alzira, Spain), as the plasma activity of AT assessment program was initiated. In total, 1352 patients were evaluated for screening of which 84 patients declined to participate for sampling of plasma levels AT and future research, or were not able to consent because of their clinical conditions. Additionally, 643 patients did not meet the eligibility criteria. Screening, eligibility and enrolment of patients are shown in **Figure 1**.

Inclusion criteria were being male or female patients, at least 18 years of age undergoing elective heart surgery. To be eligible, subjects had to present a baseline AT activity <120% and >60% and willing to comply with all aspects of the study protocol, including blood sampling, throughout the study period.

Exclusion criteria included non-elective surgery, documented AT deficiency, history of anaphylactic reaction(s) to corticoids drugs, allergies to excipients and pregnancy. Similarly, patients with preoperative (inhaled or oral administration) or intraoperative corticoid treatment were excluded. Patients scheduled for multi-valvular, combined surgery (not exclusively valvular) and aortic valve repair procedures were cause for exclusion. The pre-CPB use of intra-aortic balloon pump or ventricular assist device, were also excluded.

#### Study Interventions

The study cohort (treated DX group; DG) of subjects consisted of adult patients scheduled to undergo elective and isolate aortic valve replacement surgery with CPB at La Ribera University Hospital, using a mini-sternotomy surgical technique and DX, as a part of the initial ERAS program applied between May 2013 and February 2016. As of this latter date, ERAS program expanded their clinical implementations, thus rendering a comparative study unworthy.

The investigational drug (DX disodium phosphate, Fortecortin; ERN S.A., Barcelona, Spain) was administered immediately before anesthesia induction as a single dose (4–8 mg i.v., according to patient weight <80 kg or >80 kg, respectively).

The control cohort (untreated DX group; CG) involved those patients who underwent the same procedure by complete sternotomy surgical technique with no use of DX, before the inclusion of these two clinical implementations, from December 2011 to May 2013.

Patients scheduled for coronary artery bypass grafting were not included in this study, given the methodological bias that the use of CPB (“on pump”) implied before the ERAS program was initiated. Nevertheless, they were –marginally- considered to evaluate the CPB impact on AT activity. 154 coronary patients were performed “on pump” or with CPB (C CPB+) and 53 coronary patients were scheduled “off-pump” or without CPB (C CPB−).

#### Data Collection and Time Points

For each patient, sociodemographic pre-CPB variables, medical history, co-morbidities, risk stratification according to the EuroSCORE and intra-operative details (e.g., types of surgical interventions, CPB and aortic cross-clamp times, heparin and protamine dose, transfusion’ requirements) were recorded. The length of ICU and hospital stay and outcomes at discharge, were also collected. Mortality up to 28 days until discharge was documented.

Laboratory data included blood count, biochemistry (i.e., serum creatinine and liver enzymes) and coagulation tests (i.e., AT activity, activated partial thromboplastin time, prothrombin time, international ratio and fibrinogen).

Throughout the study clinical data were collected at five separate time points: preoperatively, day before surgery (AT1), during CPB (AT2), at ICU admission (AT3) and (AT4) at 12 h and (AT5) at 24 h post-intervention, respectively.

#### Study Outcome: Assessment of Efficacy

The primary efficacy endpoints of the study were AT activity levels at different time points higher in DG *vs.* CG in order to determine whether DX treatment could be an efficacious strategy to avoid postoperative decreased AT levels. Secondary efficacy endpoints were the length of stay in ICU and hospital.

Additional secondary endpoints were related to the postoperative complication rate (safety outcomes). These secondary endpoints included surgical re-exploration (due to bleeding), low cardiac output syndrome (need for inotropic support > 48 h), myocardial infarction, adverse neurologic outcome, acute renal failure (peak serum creatinine > 2 mg/dl, or twice the baseline), thromboembolic events (myocardial infarction, stroke, mesenteric infarction, or peripheral or pulmonary thromboembolism) and intra-hospital mortality.

#### Cardiopulmonary Bypass Procedures

Details of anesthesia and CPB equipment and technique were carried out as described previously (Muedra et al., 2011). Briefly, after anesthesia (etomidate or propofol, remifentanil, and rocuronium) CPB was established through a mini-sternotomy approach (in DG) or standard median sternotomy (in CG), aortic root cannulation and single or bi-cava atrial cannulation for venous return. The circuit priming volume before beginning CPB was 600 ml. Antegrade/retrograde intermittent cold blood cardioplegia (4:1) was used. The pump flow was set at 2.4–2.6 l/m2 per min and a target mean arterial pressure set at 65–70 mmHg. Body temperature during CPB was maintained between 35.0 and 35.8°C (mild hypothermia). All patients received tranexamic acid intraoperatively (20 mg/kg body weight intravenously before the induction of anesthesia, 1 mg/kg body weight/min during CPB and finally, 20 mg/kg body weight after the protamine dose). Active Coagulation Time (ACT) was determined immediately after the induction of anesthesia [3 min after the loading heparin dose (300 IU/kg body weight), 5 min after the initiation of CPB and subsequently every 15 min]. An ACT of 460 s or greater was considered satisfactory. A specific perioperative transfusion algorithm was applied to maintain haematocrit above 25%, according to the clinical condition, hemodynamic status, need for inotropic support and age of the patient (Muedra et al., 2011). After the operation, all patients were released to the ICU. The clinical course of patients and post-surgery complications were followed until discharge. Insulin in continuous perfusion according to our institutional protocol was established to maintain glycaemia between 100 and 150 mg/dl.

To prevent nausea and vomiting postoperative (NVPO), all patients – control and study group- received ondansetron (4 mg i.v.) in the 30 min before the end of the procedure; prior to anesthesia induction, patients in the DG exclusively, received DX (4-8 mg i.v.).

The DX administration and mini-sternotomy surgical approach were selected to initiate our ERAS program as main clinical implementations. From February 2016, the ERAS program was completed with other implementations.

### Sampling Manipulation

Five milliliters of blood were withdrawn into Vacutainer Tubes (Becton Dickinson, Rutherford, NJ, United States) containing 3.8% sodium citrate (9:1 v/v). Samples were immediately centrifuged at 3000*g* for 15 min at room temperature. The plasma fraction was transferred to polypropylene tubes and frozen for weekly analysis.

### Assessments

The AT plasma activity (expressed in percentage, normal range, 60–120%) was determined at the central hospital laboratory. The automated chromogenic anti-FXa assay for the quantitative determination of functional AT was Innovance AT Test designed for use on BCS XP SYSTEM haemostasis analyser (Siemens Healthcare Diagnostics Ltd., Erlange, Germany). Data were expressed as mean ± SE.

### Determination of SERPIN Expression in Endothelial Cells by qRT-PCR

Human aortic endothelial cells (hAEC), human coronary artery endothelial cells (hCAEC) and human cardiac microvasculature endothelial cells (hCMEC) from Promocell (Heidelberg, Germany) were seeded (50000 cells/plate) and grown until 80% confluence.in endothelial basal medium (EBM, Promocell) supplemented with fetal calf serum (FCS; 50 mL/mL), human basic fibroblast growth factor (hbFGF; 10 ng/mL), human vascular endothelial growth factor (hVEGF; 0.5 ng/mL), human epidermal growth factor (hEGF; 5 ng/mL), human recombinant insulin-like growth factor-1 (R3IGF-1; 20 ng/mL), ascorbic acid (1 mg/mL) and hydrocortisone (0.5 mg/mL), (all of them from Promocell; Heidelberg, Germany), 0.015 mg/mL amphotericin B (Biowhittaker; Lonza Basel, Switzerland) and 30 mg/mL gentamicin (Genta-Gobens; Laboratorios Normon SA, Tres Cantos, Madrid, Spain). Then, cells were incubated overnight in EBM with hbFGF (10 ng/mL), hVEGF (0.5 ng/mL), hEGF (5 ng/mL), R3IGF-1 (20 ng/mL), ascorbic acid (1 mg/mL) 0.015 mg/mL amphotericin B and 30 mg/m and gentamicin, but without hydrocortisone. To ensure corticoids from the medium did not interfere, charcoal stripped FCS was used instead of FCS. Cells were treated with DX 0 nM (vehicle) or 500 nM for 0, 1, 3, and 24 h and lysated in TRIpure^®^ reagent (Invitrogen^TM^). Total RNA was obtained as previously described (Montó et al., 2012) quantified and analyzed by running 1 μg of each sample by microfluidic electrophoresis using the Experion^TM^ automated electrophoresis system (Bio-Rad, Madrid, Spain) following the manufacture’s conditions. Reverse transcription reaction was performed with 2 mg of total RNA using InPromII Reverse Transcriptase (Promega Biotech Iberica, Spain) and (dT)16 as primer, following the manufacturer’s instructions. Quantitative PCR was made using TaqMan fluorescent probes for *SERPINC1* and GAPD as reference, according to the manufacturer’s instructions (Applied Biosystems, United States). Relative quantification was obtained by the 2^−ΔΔCT^ method. We analyzed (in duplicate reactions) a 10-fold dilution of the RT reaction of each sample using the TaqMan^TM^ Gene Expression Assays (Applied Biosystems, United States). Each treatment was done in triplicate wells (triplicate data were averaged) with experimental “*n*” corresponding to different experiments.

### Migration of Human Endothelial Cells

Cell migration was assessed using a “scratch wound healing assay” (Staton et al., 2004; Guo et al., 2014). 50.000 cells per plate were seeded in 24 well-plates (Costar, Corning, NY, United States) and cultured until 80–90% confluence, at 37°C and 5% CO_2_, in EBM, supplemented with FCS (50 mL/mL), hbFGF (10 ng/mL), hVEGF (0.5 ng/mL), hEGF (5 ng/mL), R3IGF-1 (20 ng/mL), ascorbic acid (1 mg/mL), hydrocortisone (0.5 mg/mL), amphotericin B (0.015 mg/mL), and gentamicin (30 mg/mL). Then, cells were incubated overnight for serum starvation in EBM and antibiotics (working medium), except hCMECs, which were supplemented with FCS 0.5 mL/mL to avoid cellular damage. Then, the cell monolayer was scraped in a straight line with a sterile p1000 pipette tip. The media was aspirated and replaced with the same media to remove non-adherent cells. Light microscope images (Leica DM IL LED, 2.5× magnification) were obtained at incubation start time, (time 0) and again after 6 h of incubation in fresh working medium supplemented with AT 0 IU/mL (vehicle), 0.5 IU/mL or 1 IU/mL (Grifols, Spain). Non-populated scratch areas were quantified by ImageJ freeware (NIH Image) and the per cent closure was calculated by measuring the filled area in presence of AT *vs.* control. Each treatment was done in duplicate wells (duplicate data were averaged) with experimental “*n*” corresponding to different experiments.

### Angiogenesis of Human Endothelial Cells

The capacity of hAEC, hCAEC, and hCMEC to form capillary tubule-like networks was tested by seeding 60.000 cells in 96-well plates (Costar, Corning, NY, United States) pre-coated with 50 mL growth factor reduced Matrigel^®^ (BD Biosciences Bedford, MA, United States) as previously described (Staton et al., 2004; Guo et al., 2014). The cells were incubated 18 h at 37°C and 5% CO_2_, in EBM supplemented with FCS (50 mL/mL), hbFGF (10 ng/mL), hVEGF (0.5 ng/mL), hEGF (5 ng/mL), R3IGF-1 (20 ng/mL), ascorbic acid (1 mg/mL), hydrocortisone (0.5 mg/mL), 0.015 mg/mL amphotericin B, and 30 mg/mL gentamicin and in presence of AT 0 IU/mL (vehicle), 0.5 IU/mL or 1 IU/mL. At the end of the incubation time, a final fluorescence cell staining was performed by incubating cells for 15 min with 5 mM calcein AM (Invitrogen; Molecular Probes Inc., Paisley, United Kingdom). The images were visualized using a Leica DM IL LED microscope coupled to a Leica fluorescence camera at appropriate magnification (25×). Total number of tubules in each visual field (four different fields for plate) were quantified using ImageJ freeware. Each treatment was carried out in duplicate wells (duplicate data were averaged) with experimental “*n*” corresponding to different experiments.

### Arterial Ring Model of Angiogenesis

Male Wistar rats (270–300 g) bred in our animal facility were anesthetized with isoflurane and killed by decapitation. Segments of rat thoracic aorta of were aseptically removed and cleaned from adipose tissue as previously described (Vicente et al., 2016). Aortic rings (1 mm) were sectioned in a Petri dish on ice and rinsed with five consecutive washes of Krebs solution (NaCl 118 mM, KCl 4.7 mM, CaCl_2_ 1.8 mM, KH_2_PO_4_ 1.2 mM, NaHCO_3_ 25.0 mM, glucose 11.0 mM). The periaortic fibroadipose tissue was removed with fine micro-dissecting forceps and scissors, carefully avoiding damage to the arterial wall. 50 μl of Matrigel^TM^ per well were added to 96-well plates, which were kept on ice until use and the arterial rings were randomized into wells. After 15 min at room temperature once Matrigel^TM^ had polymerized, 200 μl of EBM medium (Promocell, Heildelberg, Germany) were added to each well. Media were supplemented with 0.015 μg/mL Amphotericin B, 30 μg/mL Gentamicin and FCS 50 μL/mL. Plates were incubated at 37°C and 5% CO_2_ for 6 days, renewing the medium, as well as the stimuli required, the day after starting the experiment and every 2 days thereafter. Experiments were performed in duplicate with at least three different animals. A control group, consisting of aortic rings dissected from the same aorta without any treatment, was included in each experiment to minimize inter-assay variability.

At the beginning of the experiment, processing parameters were fixed and all images were collected by the same researcher and under the same observation conditions (light, contrast, and magnification). The cultures were photographed daily from day 3 to day 6 using an inverted microscope Leica DM IL LED coupled to a Leica Digital Camera, at appropriate magnification (25×). The length of the longest vessel sprouting from the rat aortic rings was measured as the distance in the *x-* and *y*-axis, (taking the outer surface of the ring as the starting point) using Leica Microsystems LAS Software V3.7.0 (Heerbrugg, Switzerland) as has been described elsewhere (Vicente et al., 2016).

### Statistical Analysis

After assessing normality of data (quantitative variables), bivariate analysis was carried out using the Student *t*-test, Welch test or Dunnet’s test as appropriate.

Bivariant analysis of nominal variables such as gender, re-intervention, mortality and major adverse cardiac events (MACE) according to corticosteroid treatment, were performed with chi-square or Fisher exact test as appropriate. A Dunnet’s test was also performed to compare pre-operative AT levels with AT values measured during and at different times after surgery. Significance was defined as *p* < 0.05.

## Results

### Consequences of Dexamethasone Administration on AT Activity During CPB

As previously reported, our research group has focused its interest in the analysis of AT plasma activity and its correlation with the postoperative outcomes in patients undergoing cardiac surgery.

Based on this fact, a total of 1352 patients were retrospectively screened from January 2011 to February 2016 at La Ribera University Hospital (Alzira, Valencia. Spain) (**Figure 1**).

The cohort comprised 51 patients in the control group (CG) and 29 patients in the group receiving dexamethasone (DG). **Table 1** summarizes the findings when the variables listed were compared according to steroid treatment. Differences between the two groups were detected for some variables.

**Table 1:** Sociodemographic, AT activity and clinical characteristic of patients included in the study.

	Replacement aortic valve surgery		
	Control group, CG (dexamethasone untreated)	Dexamethasone group, DG (dexamethasone treated)	Significance (bilateral)
Patients	*n* = 51	*n* = 29	
Male gender	22 (44%)	14 (50%)	0.331
Age (years)	70.39 ± 10.05	71.07 ± 11.53	0.785
BMI (kg/m^2^)	30.23 ± 5.66	30,33 ± 4.14	0.929
COPD	4 (8%)	3 (10.71%)	0.545
Diabetes on medication	12 (24%)	6 (21.43%)	0.687
Hypercholesterolemia	18 (36%)	10 (35.71%)	0.895
Hypertension	37 (74%)	19 (67.86%)	0.287
Serum creatinine (mg/dL) preoperative	1.11 ± 0.59	1.03 ± 0.29	0.438
Hemoglobin (mg/dL) preoperative	12.11 ± 1.48	11.92 ± 2.18	0.072
Logistic EuroSCORE I	4.84 ± 2.40	6.37 ± 4.14	0.075
Anesthesia time (min)	153.5 ± 26.21	195.3 ± 37.83	<0.001^∗∗∗^
CPB time (min)	68.86 ± 12.99	93.17 ± 25.39	<0.001^∗∗∗^
Aortic cross-clamp time (min)	43.43 ± 10.17	71.62 ± 21.28	<0.001^∗∗∗^
ACT activity	155.21 ± 0.82	150.6 ± 3.51	0.387
**Antithrombin activity (%)**		
Prior surgery (AT1)	97.08 ± 14.36	93.31 ± 10.42	0.219
During CPB (AT2)	58.12 ± 9.11^##^	67.41 ± 10.49^##^	<0.001^∗∗∗^
ICU admission (AT3)	64.53 ± 9.34^#^	65.83 ± 11.16^##^	0.579
12 h after surgery (AT4)	75.84 ± 11.83^##^	76.00 ± 10.33^##^	0.953
24 h after surgery (AT5)	81.69 ± 13.10^##^	84.55 ± 10.47^#^	0.317

*Data represent the mean ± SE. A Dunnet’s test was also performed to compare AT levels pre-operative with AT values measured during and at different times after surgery. Significance was defined as ^#^p < 0.05, ^##^p < 0.01 *vs.* preoperative level; and as ^∗^p < 0.05, ^∗∗^p < 0.01, ^∗∗∗^p < 0.001 *vs.* comparing between groups. AT, Antithrombin (%); ACT, Active Coagulation Time (min); BMI, body mass index (kg/m2); COPD, chronic obstructive pulmonary disease; EuroSCORE, European System Cardiac Operative Risk Evaluation*.

Mean preoperative active coagulation time (ACT) was 155.2 ± 0.8 in the CG and 150.6 ± 3.5 in the DG. Mean preoperative AT activity (AT1) was within the normal range (80–120%), with a mean value of 97.08 ± 14.36% in the CG and 93.31 ± 10.42% in the DG. Mean AT activity during CPB (AT2), at ICU admission (AT3) and at 12 and 24 h post-intervention (AT4, AT5, respectively) significantly dropped in both groups (*p* < 0.001) respect to basal level (AT1) and remained significantly reduced throughout the study period (24 h). This difference in AT activity was attenuated at 24 h (AT4) respect to the basal value in the DG (*p* < 0.05). These data are summarized in **Table 1**.

**FIGURE 2:**
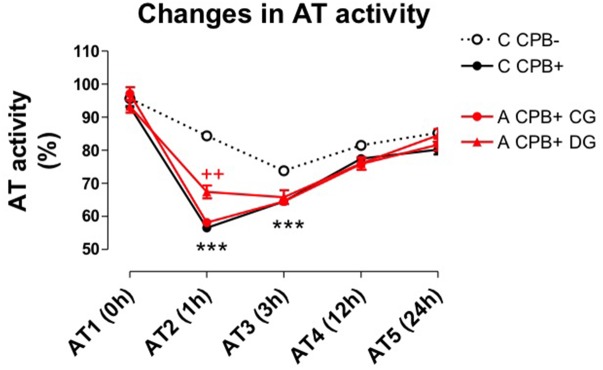
Evolution of AT activity measured preoperatively (AT1), during CPB (AT2), at ICU admission (AT3) and at 12 and 24 h post-surgical time (AT4 and AT5, respectively). Data are expressed as the mean ± SE; ^∗∗^*p* < 0.001 *vs.* C CPB– group; ^++^*p* < 0.01 *vs.* A CPB+ CG (2 way ANOVA, Graph Pad software). C, coronary patient; A, aortic patient; “on pump” or with CPB procedure (CPB+); “off-pump” or without CPB procedure (CPB–); dexamethasone untreated, CG; dexamethasone treated, DG.

The decrease of AT2 activity level in CG (58.12 ± 9.11) was significantly higher than in DG patients (67.71 ± 10.49), *p* < 0.001. Postoperative AT activity increased without fully recovering the preoperative values. All these data and the CPB impact over AT levels in different clinical setting are shown in **Figure 2**.

On average, aortic cross-clamp time (ischemic time, measured in minutes) was higher in the group treated with DX (71.62 ± 21.28) than in controls group (43.43 ± 10.17), *p* < 0.001; and similarly mean CPB times were also higher in the DG than in the CG (93.17 ± 25.39 *vs.* 68.86 ± 12.99, respectively), *p* < 0.001. According to the previous times, the anesthesia procedure times were significantly higher in the DG than in CG (195.3 ± 37.8 *vs.* 153.5 ± 26.2; *p* < 0.001).

Outcomes variables are shown in **Table 2**. In all cases, we could not detect statistical differences between any of those variables and corticosteroid treatment. Nevertheless, hospitalization time was considerably shorter (*p* = 0.014) in DG (7.59 ± 4.08 days) than in CG (13.59 ± 16.00 days). The length of stay in ICU was 3.34 ± 0.17 *vs.* 6.19 ± 3.04, but it was not statistically significant. Postoperative cardiac output was significantly reduced (*p* < 0.05) in CG (**Table 2**).

**Table 2:** Incidence of post-surgery outcomes variables.

	Replacement aortic valve surgery		
	Control group (Dexamethasone untreated) *n* = 51	Study group (Dexamethasone treated) *n* = 29	*p*-value
Re-hospitalization	7 (14%)	5 (8.93%)	0.332
Re-intervention	2 (4%)	1 (1.79%)	0.458
Low cardiac output	7 (14%)	0 (0%)	0.002^∗∗^
Perioperative AMI	1 (2%)	0 (0%)	0.227
Arrhythmia	19 (38%)	9 (16.07%)	0.161
Lung dysfunction	2 (4%)	0 (0%)	0.361
Stroke	2 (4%)	0 (0%)	0.143
AKI	1 (2%)	5 (8.93%)	0.227
AKI in hemodialysis	1 (2%)	0 (0%)	0.495
Surgical wound infection	2 (4%)	0 (0%)	0,143
Transfusions	18 (36%)	8 (28.57%)	0.247
Mortality at day 28	1 (2%)	0 (0%)	0.343
MACE	6 (12%)	1 (3.57%)	0.105
Any complication^#^	25 (31.25%)	10 (12.5%)	0.785
ICU stay (in days)	6.20 ± 12.66	3.34 ± 3.78	0.242
Length of stay in hospitalization (in days)	13.59 ± 16.00	7.59 ± 4.08	0.014^∗∗^

*Significance was defined as ^∗^p < 0.05, ^∗∗^p < 0.01. AMI, acute myocardial infarction; AKI, acute kidney injury; Any Complication^#^: re-intervention, low cardiac output, arrhythimia, lung dysfunction, stroke, acute renal failure, mesenteric ischemia and re-hospitalization; MACE, Major Adverse Cardiac Events; ICU, Intensive Care Unit.*

On the other hand, the inclusion in the study of coronary patients scheduled for CABG, operated “on pump” (C CPB+) or “off pump” (C CPB−), allowed to observe a significant decrease of AT2 (56.50 ± 11.62 *vs.* 84.38 ± 13.67) and AT3 (64.51 ± 11.98 *vs.* 73 ± 13.61) levels, respectively. As has shown in **Figure 2**, the fall in AT activity during CPB was similar in coronary (C CPB+) and aortic (A CPB+) patients. All these findings are summarized in **Figure 2**.

### Effect of Dexamethasone on *SERPINC1* Expression in Human Endothelial Cells

**FIGURE 3:**
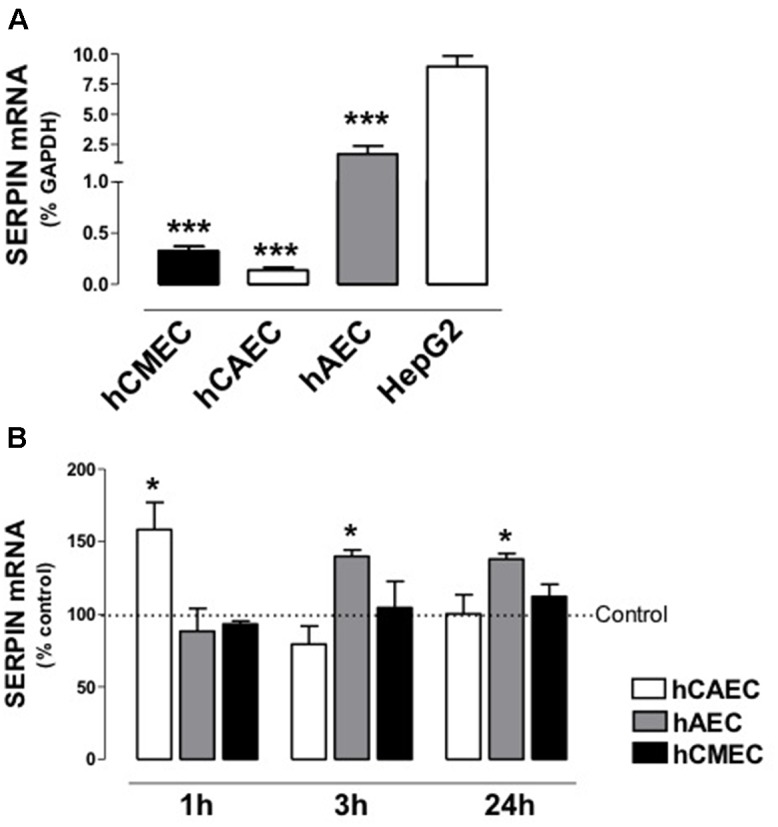
Expression of antithrombin gene (*SERPINC1*) in human endothelial cells and changes by incubation with Dexamethasone. **(A)** Expression of *SERPINC1* in human cardiac microvasculature endothelial cells (hCMEC), human coronary artery endothelial cells (hCAEC), human aorta endothelial cells (hAEC) and HepG2 human hepatoma cells. Data are expressed as mean ± SE, *n* = 5. ^∗∗∗^*p* < 0.05 *vs.* control the other two cell types. Statistical analysis was performed by one way ANOVA followed by Dunnett’s Multiple Comparison Test (GraphPad Prism 4 software). **(B)** Time Course (1–24 h) of *SERPINC1* expression in hCAEC, hAEC, and hCMEC incubated with Dexamethasone 500 nM. Data are expressed as % of the control (mean ± SE, *n* = 4–5) ^∗^*p* < 0.05 *vs.* control. Statistical analysis was performed by paired Student‘s *t*-test *vs.* control (GraphPad Prism 4 software).

*SERPINC1* expression could be detected in the three lines of human endothelial cells and in HepG2 human hepatoma cells (**Figure 3A**), being this expression more robust in HepG2 than in hAECs, hCAECs, or hCMECs. The regulation of endogenous *SERPINC1* in the three endothelial cell lines could be studied without resorting to the use of transfections of artificial reporter genes. We analyzed the effects of DX (500 nmol/L for 0, 1, 3 and 24 h) and vehicle-treated cells (control) on mRNA levels of *SERPINC1*. As demonstrated by RT-qPCR assays, treatment with DX for 1 h in hCAECs and for 3 or 24 h in hAECs, significantly increases *SERPINC1* expression (**Figure 3B**). DX did not change *SERPINC1* expression in hCMECs.

### Role of AT on Migration and Angiogenesis

**FIGURE 4:**
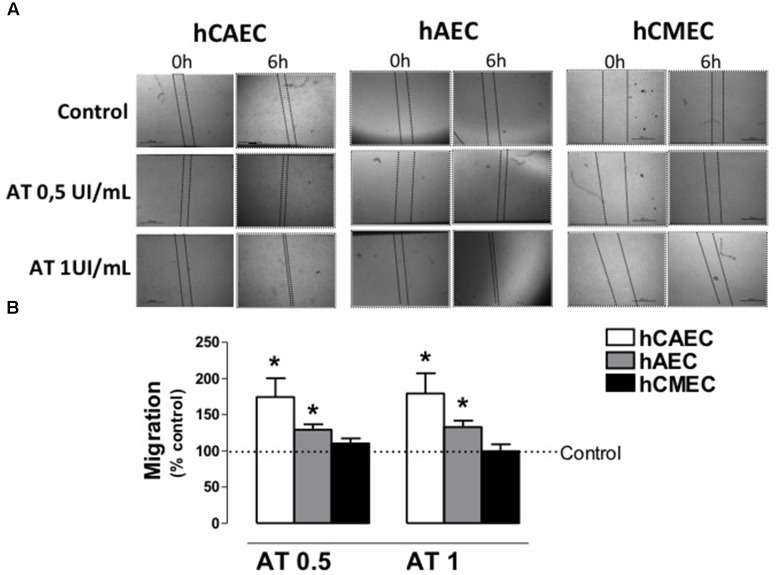
Antithrombin activity on migration of human endothelial cells. **(A)** Representative images of hCAEC, hAEC and hCMEC with scratch wounds at 0 and 6 h of incubation with different concentrations of antithrombin 0 (control), 0.5 and 1 IU/mL. **(B)** Evaluation of the migration of hCAEC, hAEC, and hCMEC incubated with antithrombin 0.5 and 1 IU/mL and calculated as % of the control (0 IU/mL). Data are expressed as the mean ± SE, *n* = 5–6 experiments performed in duplicate.^∗^*p* < 0.05. Statistical analysis was performed by paired Student‘s *t*-test *vs.* control (GraphPad Prism 4 software).

The capacity hAECs, hCAECs, and hCMECs to migrate was observed using the “scratch wound healing assay.” As shown in **Figure 4A**, 6 h after the scratch, a significant number of cells had migrated to refill the wounded area that, at this time, was 82.12 ± 2.79% of the original wound area in hCAECs, 73.03 ± 4.90% in hAECs and 66.59 ± 3.16% in hCMECs (*n* = 5). When hAECs and hCAECs were incubated with AT 0.5 IU/mL or 1 IU/mL for 6 h, the percentage of cells re-populating the original wound area relative to untreated cells (control) was significantly increased (**Figure 4B**). However, AT dis not cause significant changes in the migration of hCMECs.

**FIGURE 5:**
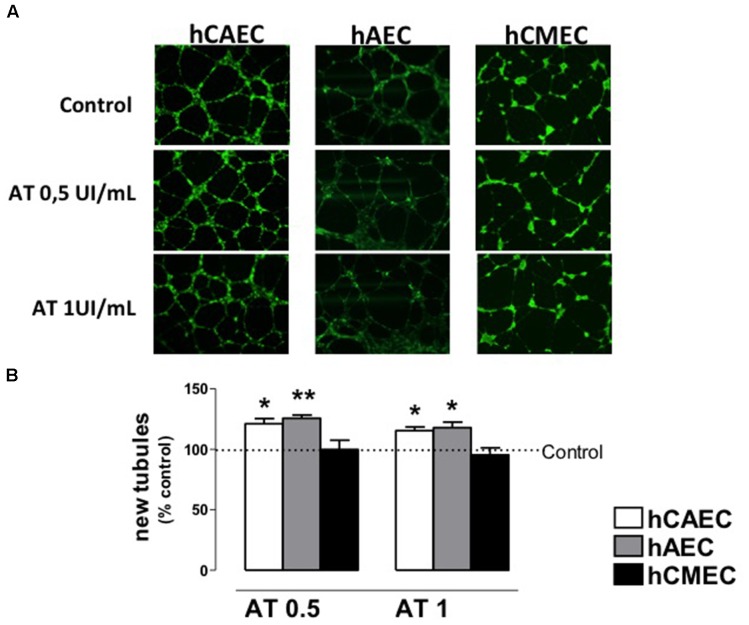
Antithrombin activity on vessel formation by human endothelial cells. **(A)** Representative images of vessel formation by hCAEC, hAEC, and hCMEC after 18 h of incubation in Matrigel with antithrombin 0 (control), 0.5 and 1 IU/mL. Fluorescent images were obtained after 5-mM calcein AM of cells. **(B)** Quantification of the new vessel formation of hCAEC, hAEC, and hCMEC incubated with antithrombin 0.5 and 1 IU/mL and calculated as % of the control (0 IU/mL). Data are expressed as the mean ± SE, *n* = 3–5 experiments performed in duplicate. ^∗^*P* < 0.05, ^∗∗^*p* < 0.01. Statistical analysis was performed by paired Student‘s *t*-test *vs.* control (GraphPad Prism 4 software).

The ability of AT to promote angiogenic activity was examined in a capillary tube formation assay. As shown in **Figure 5A**, incubation in Matrigel^®^ induced hAECs, hCAECs, and hCMECs to differentiate and form capillary tubes. To determine the activity of AT in this process, we incubated cells with two concentrations of AT 0.5 IU/mL or 1 IU/mL. Number of tubules per microscopic field in plates treated with AT are expressed as percentage relative to values in untreated plates. Treatment with AT (0.5 or 1 IU/mL) increased tubule formation in hAECs and hCAECs, but not in hCMECs (**Figure 5B**).

**FIGURE 6:**
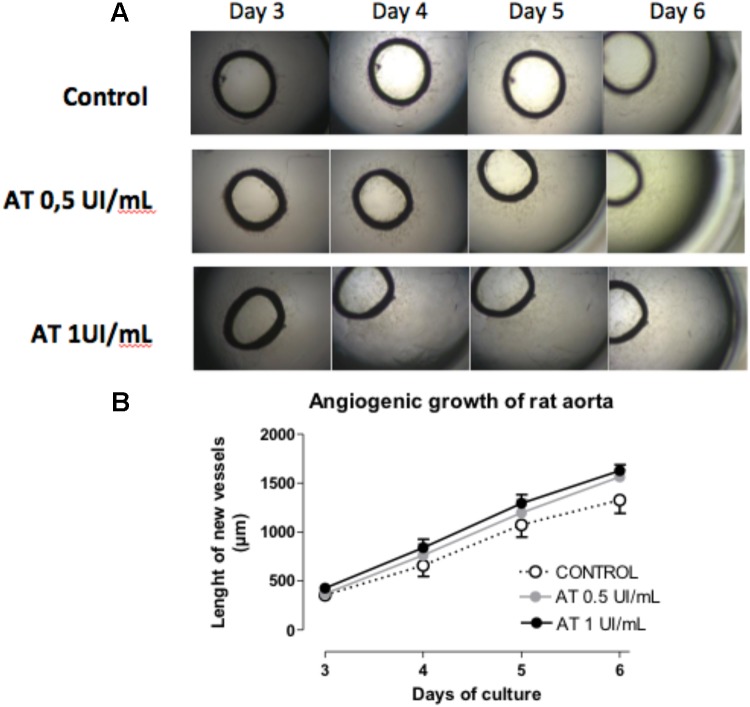
Antithrombin activity on angiogenic growth of aortic rings. **(A)** Representative images of the neovessel sprouting at days 3–6 of culture of rat aortic rings in Matrigel incubated with antithrombin 0 (control), 0.5 and 1 IU/mL. **(B)** Quantification of neovessels length along time Statistics were performed by 2-way ANOVA (GraphPad Prism 4 software). AT treatment in significantly increased angiogenic growth along time (^∗^*p* < 0.05).

The pro-angiogenic activity of AT was confirmed in an “*ex vivo*” model of arterial ring angiogenesis using freshly dissected rat aortas cultured in presence of AT 0 (control), 0.5 and 1 IU/mL. In these conditions, networks of newly formed vessels that sprouted from the arterial wall were observed after 3 days in culture (**Figure 6A**). Aortic cultures were daily photographed (from days 3 to 6 of culture). As **Figure 6B** shows, angiogenic sprouting was significantly potentiated by AT 0,5 IU/mL and AT 1 IU/mL.

## Discussion

Cardiopulmonary bypass in cardiac surgery is associated with pronounced endothelial activation, which is a key process in the development of an inflammatory and pro-coagulant response (Schmid et al., 2006; Zhang et al., 2013). Recently, the protective effect of a newly developed fucose-deficient recombinant antithrombin against histone-induced endothelial damage has revealed –in a sepsis experimental model-, that AT could target primarily the vascular endothelium (Iba et al., 2018). Our study shows further evidence of the increased expression of *SERPINC1* by DX in human endothelial cells, increasing AT levels. This fact supports the clinical evidence that patients treated with DX exhibit significantly higher AT activity during cardiac surgery with CPB. Thus, when endogenous AT activity is decreased, DX treatment could be a pharmacological alternative to exogenous AT supplementation. In addition, our results demonstrate that AT increases endothelial cell migration, promotes capillary-like tube formation in hCECs and hAECs cells and potentiates the sprouting of new vessels from aortic rings cultured in Matrigel^®^. It is well known that migration and angiogenesis contribute to vascular repair that could be relevant in the recovery of cardiovascular procedures. However, the exact role that AT plays on the endothelium in this process is not clear.

### Dexamethasone Administration Is Related to Increase AT Activity During CPB and Decreased Hospitalization After Cardiac Surgery

Antithrombin supplementation in cardiac operations with CPB is still a subject of debate. Preoperative supplementation with concentrate AT in cardiac surgery with CPB may represent a viable strategy for decreasing the haemostatic system dysregulation, preventing heparin resistance and reducing postoperative morbidity and hospitalization time (Fernandez-Rachubinski et al., 1996).

Our results demonstrate a significant optimization of postoperative cardiac output in patients treated with DX. This finding could justify the tendency to reduce the postoperative complications and the length of stay in hospitalization, observed in this group. Previous studies indicate that pre-treatment with a single dose of corticoids improved left ventricular contractility and decreased cardiac complications like arrhythmias and/or myocardial infarction, after CPB (Chiu et al., 2005); however, in others studies, no significant differences were found (Holte and Kehlet, 2002). Our group (Barettino et al., 2015) has confirmed previous findings reporting attenuation of postoperative decrease in AT after methylprednisolone treatment prior to esophagectomy (Matsutani et al., 1998) and that DX improves the circulating levels of this serpin in patients treated with L-Asparaginase contributing to reduce the risk of severe AT acquired deficiency related to this treatment (Hernández-Espinosa et al., 2009).

As our group has previously reported, corticoids -among other components- were able to activate *SERPINC1* transcription in human hepatoma cells HepG2; DX was found to be the most potent with maximal effect around 500 nmol/L. Increased *SERPINC1* transcription was detected as early as 1 h after DX treatment and remained stable for up to 48 h (Barettino et al., 2015).

Our current results indicate that DX exhibit a similar activity on human aortic and coronary endothelial cells (hAECs and hCECs), where a significant increase in the expression of *SERPINC1* was observed after DX treatment. Extrapolating this to a clinical setting, this finding could explain the increase in AT activity found after DX treatment in patients undergoing cardiac surgery with CPB.

The present study fulfilled the primary efficacy endpoints as it shows that preoperative supplementation with DX in patients undergoing aortic valve replacement surgery minimizes the intraoperative decrease in AT activity, despite the more adverse clinical conditions in this group of patients. Effectively, the worse intraoperative conditions were established in the DG. So, the cross-clamp aortic and CPB times (and indirectly, total anesthesia time) were significantly longer in the DG than in the CG (untreated patients), probably justified by minimally invasive approach (mini-sternotomy), smaller access than conventional surgical approach (full sternotomy), that forces, a demanding learning curve for surgeons and takes longer time. Despite this, the mini-sternotomy approach has not improved clinical outcomes in hospitalization time and/or postoperative complications (Corona et al., 2015); however, it has shown significantly longer CPB times. In this clinical setting, a longer CPB time lead to higher consumption of AT and decreased AT synthesis by the liver and according to our current results, probably decreased endothelial function by the loss of the potential protective effect of AT on the vascular endothelium. It is well know, that the main pathway leading to low levels of AT activity is the continuous consumption triggered by thrombin formation during CPB in the presence of high heparin concentrations. It is, therefore, not surprising that CPB time is, among other factors (advanced patient age, procedures involving opening of the cardiac chambers), one of the main determinants of AT activity (Ranucci et al., 2005), as it happens in aortic valve replacement surgery.

In spite of these adverse scenarios, our clinical results detect differences in the postoperative outcomes of DX-treated group *vs.* control group, as a significant -and clinically very relevant- decrease in hospitalization time. Lacking the power for specific clinical outcome assessment, the length of ICU and hospital stay may be considered as a surrogate for a composite number of moderate to severe complications that result in a prolonged stay (Ranucci et al., 2005).

It is known that the use of CPB implies a significant fall in AT activity (Spiess, 2000; Chandler, 2005) and significant decreases of intra- and post-operative AT levels would have been expected in the aortic or coronary patients under CPB. In fact, our results show a significant decrease in AT activity at the completion of CPB in coronary patients compared to “off pump” coronary patients. In addition, it is interesting to remark that the fall in AT activity during CPB was similar in aortic and coronary patients suggesting that it is related to CPB procedure rather than to intervention. As expected, in coronary and aortic patients the lowest AT activity was reached during CPB (AT2) and at ICU admission (AT3). AT activity in postoperative intervals (12–24 h) did not reach the preoperative values, although maintaining lower AT levels throughout all this period. However, in the group of aortic patients under DX treatment (DG) the fall in AT activity was significantly lower during CPB time. These results observed after acute administration of DX, agree with previous observational studies, which highlighted that patients chronically treated with corticoids, and undergoing cardiac surgery, exhibit a lower fall in AT activity compared to untreated patients (Barettino et al., 2015).

### AT Activity on Endothelium

In addition to its well-established anticoagulant function, antithrombin has been shown to have antiangiogenic functions by inhibiting endothelial cell proliferation, blood vessel growth in the chick embryo and tumor growth in mice (Zhang et al., 2004), relating this action to a possible AT antitumoral activity. This antiangiogenic/antitumoral activity of AT was only observed when native AT undergoes conformational alterations induced by proteolytic cleavage (Bhakuni et al., 2016), but the role of native AT on the angiogenic process in physiological conditions has not been sufficiently studied. For this reason, to complement the understanding of the beneficial relationship observed between AT activity and clinical outcomes after cardiac surgery, we analyzed the activity of native AT on endothelial function and focus our attention to two processes, migration and angiogenesis, closely related to the recovery of the vasculature after CPB.

Firstly, we analyzed if hECs express *SERPINC1* in detectable levels. Present results show that *SERPINC1* is expressed in hAECs, hCECs, and hCMECs, with mRNA levels being higher in hAECs than in the other two cell lines. Next, we assayed AT action on migration and formation of capillary-like networks and the results obtained indicate that this activity depends on the hEC type. In primary cultures of hECs obtained from large arteries as aorta and coronary artery, AT favors migration and formation of new tubules. This activity was not observed in primary cultures of hECs from microvasculature as occurs in hCMECs. It is interesting to note that an increase in cell motility can be interpreted as a measure of an angiogenic response, as angiogenic factors are known to stimulate cell movement (Staton et al., 2004). The concordant activity of AT in both processes, migration and angiogenesis in each hEC type, reinforces the results obtained and suggest that AT activity on hECs is not uniform and depends on cell origin.

As the knowledge about endothelium increased, it became apparent that ECs in large vessels have different functions from those in the microvasculature and these differences are reflected in different phenotypical characteristics (Bouïs et al., 2001). These peculiar characteristics could explain the discrepancy in results observed in AT activity on ECs, depending on their origin from macro or microvascular network. Our results show that DX increases *SERPINC1* expression and AT promotes migration and angiogenesis in human ECs obtained from larger vessels, whereas DX and AT have no activity on human ECs from microvessels. The same lack of activity for AT, together to an antiangiogenic activity for modified AT was found by other authors in other endothelial cell lines (Larsson et al., 2001; Zhang et al., 2004, 2005; Azhar et al., 2016; Luengo-Gil et al., 2016).

To confirm the pro-angiogenic activity of AT in ECs from large vessels such as aorta, another assay was performed using rat aortic rings cultured in Matrigel^®^ (Vicente et al., 2016) and the results obtained were similar to those previously shown with hAECs. Incubation with AT potentiates the angiogenic growth by increasing the length of the capillary-like network formed.

Therefore, according to our results, the potential beneficial outcomes associated to maintain adequate levels of AT could be related, at least in part, to a protective effect of AT on endothelial function favoring migration and angiogenesis in human ECs from aorta and coronary artery. This activity could be especially relevant in patients under CPB during cardiac surgery, in which the AT could limit the damage on vascular endothelium induced by CBP. DX treatment, which increases AT expression in hAECs, could contribute to this beneficial effect.

### Limitations and Conclusion

Despite the limitations of present study, such as the small sample analyzed, its retrospective nature, potential selection bias (surgical technique), limited term of study adjusted to 24 h and the use of an empirical -untested- dose of DX, our results confirm “*in vivo*” and in clinical conditions the effect of DX on AT expression/activity observed in cells in culture. DX treatment could be a therapeutic alternative to meliorate AT activity in surgical procedures involving a decrease in AT activity. Additionally, DX supplementation will have an impact on health care costs, as it is more economical than other pharmacological options that will have an effect on AT activity.

In conclusion, our clinical study supports a role for preoperative DX supplementation in the prevention of intraoperative decreased AT activity. The novelty of our study is the preoperative use of DX aimed to avoid an excessive decrease of AT activity during CPB.

Further research is needed to assess the level of endothelial damage induced by CPB, especially in hAECs, probably through tissue biomarkers and to evaluate the potential protective effect of AT on endothelial function, correlating these aspects with morbidity in a clinical setting.

## Ethics Statement

The clinical study was set up in accordance with the Declaration of Helsinki, the Good Clinical Practice and approved by the local ethics committee from the La Ribera University Hospital. Written informed consent was obtained from all patients. The experimental procedures complied with guidelines established in Spanish legislation (Royal Decree RD 1201/2005) and were approved by the Experimental Animal Ethics Committee of the University of Valencia (Spain).

## Author Contributions

VM designed the clinical study, conducted data collection, and carried out clinical evaluation of patients in the study. PD and PP conceived and designed the experimental procedures, analyzed the data, and interpreted the results. CA, FM, and ÁB performed the experiments. VR and LM were involved in data analyses and interpretation of the results. All authors contributed to the writing and approved the final manuscript.

## Conflict of Interest Statement

The authors declare that the research was conducted in the absence of any commercial or financial relationships that could be construed as a potential conflict of interest.
